# Contact Heat Evoked Potentials in China: Normal Values and Reproducibility

**DOI:** 10.3389/fnhum.2021.747553

**Published:** 2022-01-11

**Authors:** Bo Sun, Hongfen Wang, Zhaohui Chen, Fang Cui, Fei Yang, Xusheng Huang

**Affiliations:** ^1^Neurological Department of the First Medical Center, Chinese PLA General Hospital, Beijing, China; ^2^Geriatric Neurological Department of the Second Medical Center and National Clinical Research Center for Geriatric Diseases, Chinese PLA General Hospital, Beijing, China

**Keywords:** contact heat evoked potentials, normal values, reproducibility, small fiber neuropathy, intraepidermal nerve fiber density

## Abstract

**Background:** Contact heat evoked potentials (CHEPs) is used to diagnose small fiber neuropathy (SFN). We established the normal values of CHEPs parameters in Chinese adults, optimized the test technique, and determined its reproducibility.

**Methods:** We recruited 151 healthy adults (80 men; mean age, 37 ± 14 years). CHEPs was performed on the right forearm to determine the optimal number of stimuli, and then conducted at different sites to establish normal values, determine the effects of demographic characteristics and baseline temperature, and assess the short- (30 min) and long-term (1 year) reproducibility. N_2_ latency/height varied with age and sex, while P_2_ latency/height and N_2_–P_2_ amplitude varied with age. The optimal number of stimuli was three.

**Results:** N_2_ latency/height (*t* = 5.45, *P* < 0.001) and P_2_ latency/height (χ^2^ = −4.06, *P* < 0.001) decreased and N_2_–P_2_ amplitude (*t* = −5.01, *P* < 0.001) and visual analog scale score (χ^2^ = −5.84, *P* < 0.001) increased with increased baseline temperature (35 vs. 32°C). CHEPs parameters did not differ with time (baseline vs. 30 min vs. 1 year).

**Conclusion:** We established normal CHEPs values in Chinese adults. We found that CHEPs parameters changed with baseline temperature and that the short- and long-term test reproducibility were satisfactory.

## Introduction

Small fiber neuropathy (SFN) is a peripheral neuropathy that affects small-caliber thinly myelinated Aδ and unmyelinated C fibers (McCarthy et al., 1995; Said, 2003; Hoitsma et al., 2004). No consensus yet exists on the standard diagnostic criteria for SFN. Some studies report that intraepidermal nerve fiber density (IENFD) assessment is the most reliable diagnostic test for SFN (Hoitsma et al., 2004; Lauria et al., 2010a). Other studies use a combination of sensory and autonomic symptoms (not otherwise explainable) as well as intact large-fiber function on examination (normal vibration sense and nerve conduction studies) with abnormal IENFD and/or abnormal temperature threshold testing; if any two of these criteria are met, then SFN can be diagnosed (Lauria et al., 2010b; Tesfaye et al., 2010). However, IENFD measurement requires skin biopsy and immunohistochemical staining, and the sensitivity of IENFD for SFN depends largely on the chosen cut-off values (Nebuchennykh et al., 2009). Furthermore, although this test has been widely applied in Western countries, it is not commonly available in China. Temperature threshold testing requires subject cooperation, although standardized protocols have been published, the technique varies among laboratories. Some experts’ reviews highlighted the role of neurophysiology in the diagnostic work-up of SFN (Terkelsen et al., 2017). Therefore, novel, simple, and standardized diagnostic tools of neurophysiology for SFN are required in China.

Contact heat evoked potential (CHEP) is a neuroelectrophysiologic technique in which heat stimuli consisting of rapid changes in temperature (70°C/s) are applied to the skin to evoke cerebral electroencephalographic responses conveyed by Aδ fibers (Lagerburg et al., 2015; Granovsky et al., 2016). The recorded cortical responses—namely N_2_ latency, P_2_ latency, and N_2_–P_2_ amplitude—can be used to appraise the function of small peripheral nerve fibers (Chen et al., 2006; Chao et al., 2007; Kramer et al., 2012a,^2013^). The CHEP test is objective and simple, and it is a safer approach than laser-evoked potentials for evaluating small peripheral nerve fibers (Le Quesne et al., 1990; Magerl et al., 1999; Le Pera et al., 2002; Truini et al., 2005; Chao et al., 2008). In SFN, CHEPs are sensitive and correlate with IENFD (Chen et al., 2001; Le Pera et al., 2002; Atherton et al., 2007; Chao et al., 2010; Casanova-Molla et al., 2011). Furthermore, CHEPs are not only used in the diagnosis of SFN, but also in spinal disorders (Kramer et al., 2012a).

However, to diagnose SFN using CHEPs, the establishment of normal CHEP values is vital. A CHEP study of 35 normal controls was performed in Taiwan (Chen et al., 2006), and another study established normal CHEP values in a Dutch population of 97 subjects (Lagerburg et al., 2015). Furthermore, a multicenter study determined normal CHEP values in 226 subjects from Brazil, Israel, Japan, Spain, and the United States (Granovsky et al., 2016), and a Swiss study identified CHEP normative data in cervical dermatomes in 101 healthy subjects (Jutzeler et al., 2016). However, different studies have revealed conflicting relationships of CHEP parameters with variables such as gender, age, and height (Chen et al., 2006; Lagerburg et al., 2015; Granovsky et al., 2016). Moreover, no large-scale studies of normal CHEP values have been conducted in China, where the diagnosis of SFN is restricted.

Therefore, it is necessary to establish normal CHEP values in the Chinese population. Furthermore, different studies have used differing numbers of CHEP stimuli (Atherton et al., 2007; Chao et al., 2007, 2008, 2010; Casanova-Molla et al., 2011; Lagerburg et al., 2015; Granovsky et al., 2016). In this study, we aimed to determine the optimal number of stimuli to avoid habituation and simplify the CHEP test. In addition, it is meaningful to study the reproducibility of the CHEP test and baseline temperature on the results obtained.

In this study, we aimed to optimize the CHEP testing technique (to determine the optimal number of stimuli), determine the short-term and long-term reproducibility of the CHEP test, and establish the normal values of CHEP parameters in a large sample of healthy Chinese adults.

## Materials and Methods

### Participants

Healthy participants were recruited from hospital volunteers and through advertisements between November 20, 2014 and December 31, 2016. All participants underwent neurological examinations, laboratory examinations, and nerve conduction studies (NCS) at outpatient departments in a comfortable, temperature-controlled room. Neurological examinations and NCS were performed by two experienced neurologists. Laboratory examinations were performed by two clinical laboratory physicians of Chinese PLA General Hospital. To be eligible for inclusion in the study, participants were required to meet the following criteria: (1) age ≥ 18 years; (2) no sensory symptoms or signs on neurological examination; (3) normal results on laboratory tests; (4) normal results on NCS; (5) absence of diseases that may cause polyneuropathy (e.g., diabetes mellitus, metabolic syndrome, and systemic illnesses like sarcoidosis or malignancy); (6) no alcohol abuse (arbitrarily defined as drinking at least 4 international units per day) (Saunders et al., 1993) or smoking history; (7) no history of hereditary diseases (e.g., hereditary motor and sensory neuropathy, hereditary sensory and autonomic disease, and Fabry disease); (8) no history of medication with neurotoxic drugs; and (9) no skin ulceration or infection at CHEP-stimulation sites.

This study was approved by the Ethics Committee of Chinese PLA General Hospital and was conducted in accordance with the Declaration of Helsinki. Written informed consent was obtained from each participant enrolled in this study.

### Laboratory Examinations

The following laboratory tests were conducted: complete blood count, erythrocyte sedimentation rate, C-reactive protein, renal and hepatic function, lipid profile, fasting glucose, glycosylated hemoglobin, oral glucose tolerance test, folate and vitamin B12, thyroid function, antinuclear antibody, anti-extractable nuclear antigens antibody, serum protein electrophoresis, and tumor markers. The diagnoses of impaired fasting glucose, impaired glucose tolerance, and diabetes were based on the diagnostic criteria published by the World Health Organization (2006).

### Electrophysiological Examination

All participants underwent NCS. Skin temperature was maintained at 32°C or above during the examinations. NCS were performed on the median, ulnar, tibial, peroneal, and sural nerves using the Keypoint electromyography (EMG) system (Medtronic, Inc., Minneapolis, United States). The results were measured according to the normal reference values used by the EMG Laboratory of Chinese PLA General Hospital.

### Experimental Setup

CHEP stimulation was conducted at the following sites:

(1) Forearm (FA), at approximately the upper border of the distal third of the volar aspect of the right forearm (Dermatome T1),
(2) Leg (LE), above the lateral malleolus at the upper border of the distal third of the right leg (Dermatome L5),
(3) Cervical spine (C7), at the spinous process of C7 (Dermatome C3) and
(4) Thoracic spine (T12), at the spinous process of T12 (Dermatome T12).

**FIGURE 1:**
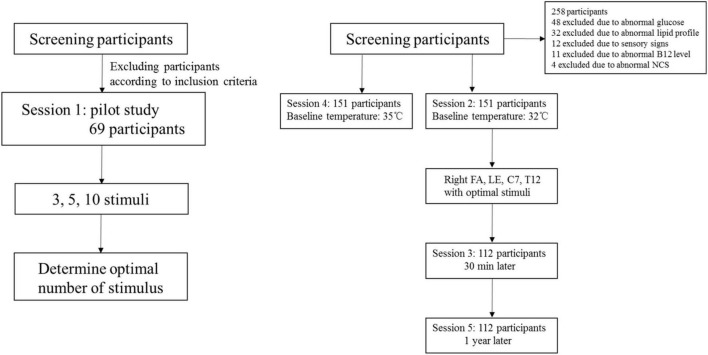
Flow chart of study design.

Five sessions of CHEP stimulation were performed (Figure 1). Session 1 served as a pilot study in which CHEP was performed on the right FA with 3, 5, and 10 stimuli to determine the optimal number of stimuli; the baseline temperature was set to 32°C. The order of 3, 5, and 10 stimuli series was performed randomly. The participants of the pilot study were not included in the main study. In session 2, CHEP was conducted on the right FA, right LE, C7, and T12, using the number of stimuli determined in session 1 and a baseline temperature of 32°C. The order of stimulation sites was randomized across participants. In session 3, CHEP stimulation was performed on the right FA with a baseline temperature of 32°C after approximately 30 min to assess its short-term repeatability. In session 4, CHEP stimulation was applied to the right FA, and the baseline temperature was increased to 35°C. The order of Session 2 and 4 were randomized. The test was performed again at the right FA with a baseline temperature of 32°C 1 year later to assess its long-term repeatability (session 5).

The visual analog scale (VAS) was used to evaluate pain intensity during the CHEP sessions. The VAS comprises a 10-cm-long horizontal line anchored by verbal descriptors of no pain (score of 0) and worst pain imaginable (score of 10). The participants were asked to place a line perpendicular to the VAS at the point that represented the average pain during CHEP stimulation.

### Contact Heat Evoked Potentials

CHEP stimulation was performed in a temperature-controlled room maintained between 20 and 24°C. A CHEP stimulator (PATHWAY, Sensory Analyzer System; Medoc Ltd., Ramat Yishai, Israel) was used to apply heat pulses at different body sites. The thermode had a diameter of 27 mm and an area of 572.5 mm^2^. It was used to deliver contact heat stimuli by increasing the baseline temperature from 32°C to a peak temperature of 51°C at a rate of 70°C/s.

Evoked potentials were recorded and analyzed using the Keypoint EMG system, which has a sensitivity of 20 μV/div and a bandpass filter of 0.1–50 Hz. The electrodes were placed on Cz and Fz according to the International 10–20 system, and referenced to linked ears. Before stimulation, all participants received 2 stimuli per body site for familiarization. The participants were instructed to keep their eyes open in a fixed, neutral position to avoid blink artifacts during the test. The participants did not know when the CHEP stimuli would be given. To avoid habituation, the thermode was moved slightly after each stimulus, which restricted in a certain area (similar distance to the recording electrode). The interstimulus interval was set between 10 and 18 s. The researchers kept the thermode in contact with the skin surface by using pressure. Two experienced researchers evaluated the recorded cortical responses and recordings with blink, muscle, or movement artifacts or other stimulation or recording interferences were eliminated. Impedance was kept below 5 kohm during the test. N_2_ latency, P_2_ latency, and N_2_–P_2_ amplitude at the four body sites were independently determined by two researchers. Disagreements between the two researchers were resolved through discussion. N_2_ and P_2_ latency were presented as “N_2_ latency/height” and “P_2_ latency/height” (latency divided by height) (Lagerburg et al., 2015).

### Statistical Analysis

Statistical analysis was performed using SPSS version 19.0 software (SPSS Inc., Chicago, IL, United States). A non-parametric test (Kruskal Wallis Test) was performed to compare CHEPs parameters (N_2_ latency, P_2_ latency, and N_2_–P_2_ amplitude) of different number of stimuli (3, 5, or 10). Logistic regression analysis was conducted to determine the relationship of CHEP parameters with age and gender, as well as their interaction. Normal values were established using the normal distribution method. The paired *t*-test and non-parametric test were performed to determine the effects of different baseline temperatures (32 vs. 35°C). Test-retest reproducibility was performed to assess the short- and long-term reproducibility of CHEP (Lagerburg et al., 2015). The data were expressed as mean ± standard deviation or median (interquartile range). A two-tailed *P-*value < 0.05 was considered statistically significant.

## Results

### Demographic Data

A total of 151 participants (80 males and 71 females) were enrolled into this study and the mean age of the participants was 37 ± 14 years (range, 21–68 years) (Table 1). All of the volunteers were of Han Chinese ethnicity. The entire examination took approximately 90 min. The CHEP procedure was well tolerated by all of the subjects. In four participants, no N_2_–P_2_ amplitudes were elicited in the case of the right LE at a baseline temperature of 32°C, but were elicited when the baseline temperature was raised to 35°C.

**TABLE 1:** Demographics, clinical and laboratory examinations of the participants.

Variable	
Number of participants	151
Examination age (mean ± SD, year)	37 ± 14 (range, 21–68)
Sex (male/female, n)	80/71
Height (cm)	168.2 ± 9.4 (range, 156–185).
Neurological examination	No sensory symptoms or signs
Laboratory examination	-
NCS	
Median motor and sensory nerve	-
Ulnar motor and sensory nerve	-
Peroneal motor and sensory nerve	-
Sural motor and sensory nerve	-

*-, negative.*

The participants were stratified into five age groups: age 20–29 years (25 males, 21 females), 30–39 years (21 males, 16 females), 40–49 years (12 males, 12 females), 50–59 years (10 males, 12 females), and 60–69 years (12 males, 10 females). As N_2_ and P_2_ latency are related to height, the data were presented as “N_2_ latency/height” and “P_2_ latency/height” (Lagerburg et al., 2015). The average height of the participants was 168.2 ± 9.4 cm (range, 156–185 cm).

### Optimal Number of Stimuli

A pilot study of 69 participants (39 males and 30 females) was performed involving CHEP stimulation of the right FA with a varying number of stimuli (3, 5, or 10). The mean age of the participants was 35 ± 12 years (range, 21–65 years). The median values of the CHEP parameters according to the number of stimuli is shown in Table 2. N_2_ latency/height (χ^2^ = 7.81, *P* = 0.056) and P_2_ latency/height (χ^2^ = 0.137, *P* = 0.934) did not significantly differ with the number of stimuli. However, N_2_–P_2_ amplitude (χ^2^ = 54.64, *P* < 0.001) significantly decreased with an increase in the number of stimuli (Figure 3).

**TABLE 2:** Results of contact heat evoked potential stimulation of the right forearm with three, five, and ten stimuli.

Parameters	3 stimuli	5 stimuli	10 stimuli	N	χ^2^	*P*
NL/H	2.11 (0.22)	2.10 (0.21)	2.10 (0.21)	69	7.81	0.056
PL/H	2.79 (0.33)	2.82 (0.35)	2.81 (0.29)	69	0.137	0.934
N_2_–P_2_	46.00 (24.70)	37.7 (26.90)	29.7 (24.00)	69	54.64	<0.001

*NL/H, N_2_ latency/height (ms/cm); PL/H, P_2_ latency/height (ms/cm); N_2_–P_2_, N_2_–P_2_ amplitude (μV); IQR, interquartile range; Values are expressed as Median (IQR).*

**FIGURE 2:**
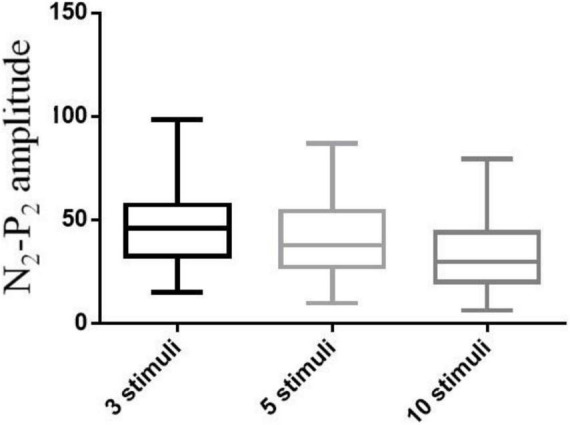
N2–P2 amplitudes at the right forearm with 3, 5, and 10 stimuli.

**FIGURE 3:**
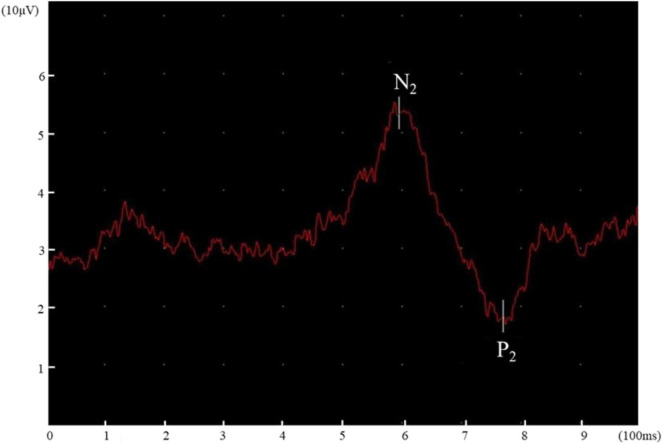
The average CHEPs trace (3 stimuli were averaged) at the right LE.

### Effects of Age and Sex on Contact Heat Evoked Potential Parameters

Regression analysis to determine the normative values of CHEP parameters showed significant effects of age and sex on N_2_ latency/height at the FA and significant effects of age on P_2_ latency/height at T12 and N_2_-P_2_ amplitude at the right LE (Table 3 and Figure 2). Therefore, we stratified the normal values of N_2_ latency/height by age and sex and those of P_2_ latency/height and N_2_–P_2_ amplitude by age at all four test sites (Table 4).

**TABLE 3:** Influence of age, sex, and the interaction of sex*age on contact heat evoked potential parameters.

Parameters	Site	Mean ± SD	*N*	*P*		
				Sex	Age	Sex*Age
NL/H	FA	2.1 ± 0.27	151	0.176	0.114	0.017
NL/H	C7	1.87 ± 0.37	151	0.295	0.024	0.104
NL/H	T12	2.13 ± 0.22	151	0.752	0.002	0.785
NL/H	LE*	2.75 ± 0.41	147	0.374	0.033	0.203
PL/H	FA	2.72 ± 0.36	151	0.468	0.221	0.155
PL/H	C7	2.56 ± 0.38	151	0.37	0.582	0.166
PL/H	T12	2.8 ± 0.31	151	0.598	0.015	0.792
PL/H	LE*	3.39 ± 0.41	147	0.787	0.71	0.577
N_2_–P_2_ amplitude	FA	42.56 ± 18.41	151	0.626	0.117	0.441
N_2_–P_2_ amplitude	C7	40.1 ± 17.25	151	0.903	0.17	0.975
N_2_–P_2_ amplitude	T12	38.02 ± 18.06	151	0.328	0.057	0.153
N_2_–P_2_ amplitude	LE*	34.94 ± 21.69	147	0.064	<0.001	0.053

NL/H, N_2_ latency/height (ms/cm); PL/H, P_2_ latency/height (ms/cm); FA, forearm; LE, leg. *In 4 patients, recordings could not be made at the leg site at a baseline temperature of 32°C.

**TABLE 4:** Normal values of contact heat evoked potential parameters in the Chinese population.

	Men								Women							

	**FA**		**C7**		**T12**		**LE**		**FA**		**C7**		**T12**		**LE**	
	**NL/H**		**NL/H**		**NL/H**		**NL/H**		**NL/H**		**NL/H**		**NL/H**		**NL/H**	

**Age (years)**	**Mean**	**Mean + 1.64S**	**Mean**	**Mean + 1.64S**	**Mean**	**Mean + 1.64S**	**Mean**	**Mean + 1.64S**	**Mean**	**Mean + 1.64S**	**Mean**	**Mean + 1.64S**	**Mean**	**Mean + 1.64S**	**Mean**	**Mean + 1.64S**
20–29	1.98	2.28	1.65	1.89	1.94	2.21	2.52	2.84	1.98	2.14	1.68	2.07	2.07	2.35	2.41	2.71
30–39	2.01	2.31	1.59	2.01	1.95	2.34	2.53	3.07	2.12	2.35	1.69	2.2	2.11	2.37	2.69	3.27
40–49	2.07	2.34	1.87	2.13	2.15	2.4	2.73	3.29	2.16	2.38	1.84	2.26	2.17	2.49	2.86	3.33
50–59	2.13	2.38	1.93	2.14	2.2	2.48	2.79	3.41	2.21	2.48	2.03	2.37	2.2	2.48	2.91	3.44
≥60	2.17	2.43	2.06	2.25	2.26	2.6	2.91	3.64	2.23	2.5	2.04	2.44	2.31	2.64	3.14	3.49

	**FA**				**C7**				**T12**				**LE**			
	**PL/H**		**N-P**		**PL/H**		**N-P**		**PL/H**		**N-P**		**PL/H**		**N-P**	

20–29	2.58	3.09	46.17	19.21	2.41	2.77	46.18	22.58	2.67	3.14	46.4	16.32	3.32	3.81	56.48	20.88
30–39	2.66	3.22	41.61	16.99	2.5	2.83	44.61	16.55	2.68	3.16	38.58	11.69	3.33	3.81	30.71	12.52
40–49	2.89	3.23	37.05	12.89	2.55	3.17	36.14	14.69	2.78	3.24	37.88	9.73	3.37	3.85	29.73	7.89
50–59	2.82	3.28	36.02	9.12	2.58	3.19	33.55	13.23	2.88	3.31	36.25	8.24	3.38	3.86	24.13	5.79
≥60	2.92	3.36	34.97	7.91	2.75	3.21	29.71	7.11	3.02	3.53	28.63	8.13	3.54	3.99	21.19	5.76

*NL/H, N_2_ latency/height (ms/cm); PL/H, P_2_ latency/height (ms/cm); N-P, N_2_-P_2_ amplitude (μV); FA, forearm; LE, leg. Values are shown as mean + 1.64 SD (NL/H and PL/H) or mean + 1.64 SD and mean – 1.64 SD (N_2_-P_2_ amplitude).*

### Effect of Baseline Temperature

**FIGURE 4:**
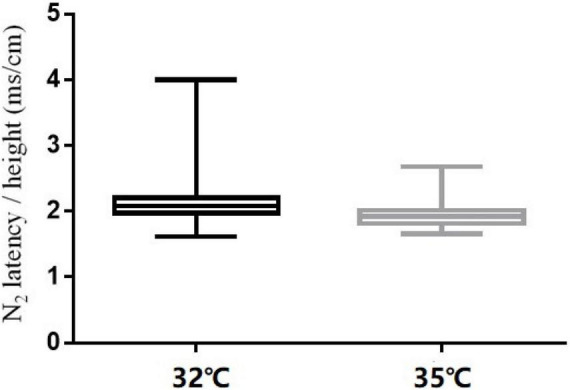
N_2_ latency/height at the right forearm for different baseline temperatures (32 and 35°C).

**FIGURE 5:**
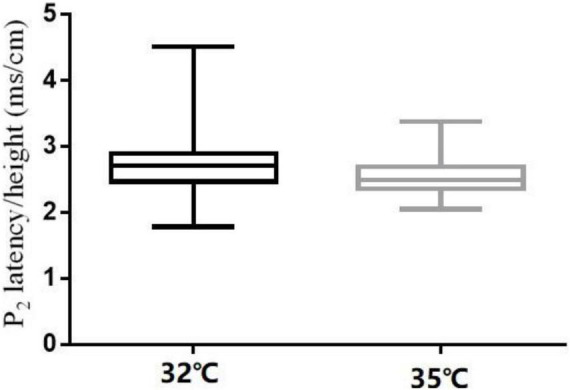
P_2_ latency/height at the right forearm for different baseline temperatures (32 and 35°C).

**FIGURE 6:**
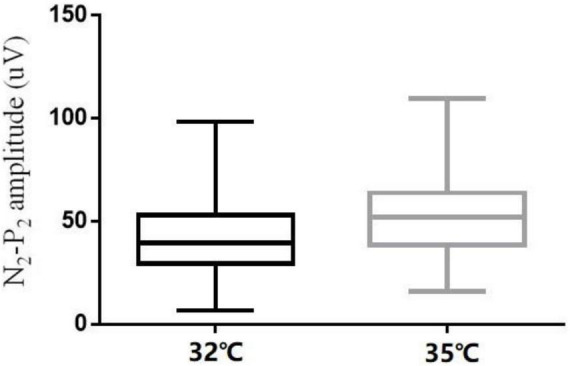
N_2_-P_2_ amplitude at the right forearm for different baseline temperatures (32 and 35°C).

The comparison of CHEP parameters at the FA and VAS scores at different baseline temperatures is shown in Table 5. N_2_ latency/height (*t* = 5.45, *P* < 0.001) and P_2_ latency/height (χ^2^ = −4.06, *P* < 0.001) significantly decreased and N_2_–P_2_ amplitude (*t* = −5.01, *P* < 0.001) and VAS score (χ^2^ = −5.84, *P* < 0.001) significantly increased with an increase in baseline temperature (Figures 4–6).

**TABLE 5:** Contact heat evoked potential parameters for the right forearm and VAS scores at different baseline temperatures.

Parameters	32°C	35°C	*N*	t(χ^2^)	*P*
NL/H	2.09 ± 0.19	1.95 ± 0.19	147	5.45	<0.001
PL/H	2.71 (0.43)	2.49 (0.34)	147	−4.06 (χ^2^)	<0.001
N_2_–P_2_	40.29 ± 18.64	53.01 ± 19.96	147	−5.01	<0.001
VAS	5 (3)	7 (3)	147	−5.84 (χ^2^)	<0.001

*NL/H, N_2_ latency/height (ms/cm); PL/H, P_2_ latency/height (ms/cm); N_2_–P_2_, N_2_–P_2_ amplitude (μV); VAS, visual analog scale; IQR, interquartile range. Values are expressed as mean ± SD or Median (IQR).*

### Reproducibility of Contact Heat Evoked Potential Parameters

A comparison of CHEP parameters at the FA during different sessions is shown in Table 6. We found no significant differences in CHEP parameters at different time intervals (baseline vs. 30 min later vs. 1 year later). The test-retest reliability of the latency/height was good.

**TABLE 6:** Test-retest reliability of contact heat evoked potential parameters for the right forearm at different time points.

Parameters	N	Test-retest reliability coefficient	Baseline vs. 30 min later	Baseline vs. 1 year later	30 min later vs. 1 year later
NL/H	112		0.718	0.7	0.717
PL/H	112		0.704	0.746	0.898
N_2_–P_2_	112		0.654	0.65	0.65

*NL/H, N_2_ latency/height (ms/cm); PL/H, P_2_ latency/height (ms/cm); N_2_–P_2_, N_2_–P_2_ amplitude (μV).*

## Discussion

This research established normal values of CHEP parameters in a large sample of healthy Chinese adults. We found that N_2_ latency/height was affected by both age and sex, while P_2_ latency/height and N_2_–P_2_ amplitude were affected by age. Three was the optimal number of stimuli, and a higher baseline temperature (35°C) could be used to elicit responses if these were not detectable at a lower baseline temperature (32°C). Furthermore, the short-term (30 min) and long-term (1 year) repeatability of CHEP testing were excellent.

In this study, sex affected the value of N_2_ latency/height at the FA, while age affected the values of N_2_ latency/height at the FA, P_2_ latency/height at T12, and N_2_–P_2_ amplitude at the LE. These findings are not completely consistent with those of previous studies (Chen et al., 2006; Lagerburg et al., 2015; Granovsky et al., 2016). A study of 35 Taiwanese subjects indicated that the values of CHEP parameters were not correlated with age, sex, and body height (Chen et al., 2006); however, the sample size of this study was relatively small, and its conclusions may not be definitive. A Dutch study of 97 healthy controls revealed that N_2_ latency/height and P_2_ latency/height were significantly correlated with age, and N_2_–P_2_ amplitude was significantly correlated with age and sex (Lagerburg et al., 2015). The authors of this study proposed that the correlation of sex with N_2_ and P_2_ latency may be attributable to height differences between men and women. A recent multicenter study of 226 healthy subjects from Brazil, Israel, Japan, Spain, and America found that women had larger amplitudes and shorter latencies than men (Granovsky et al., 2016). However, body height was not an influencing factor in their study, and therefore, it is unclear whether the above differences in CHEP parameters were caused by sex-specific differences in body height. Moreover, in the above study, age had a significant influence only on CHEP parameters at the L1 and C7 sites, and sex had a significant influence only on P_2_ latency in the leg (Granovsky et al., 2016).

Thus, different studies have revealed conflicting relationships of CHEP parameters with age, sex, and height (Truini et al., 2005; Chen et al., 2006; Chao et al., 2007; Lagerburg et al., 2015; Granovsky et al., 2016) possibly due to differences in sample size, ethnicity, and parameter processing method. However, we conducted CHEP tests in 151 healthy Chinese subjects and established normal CHEP values for the Chinese population, which is vitally important in the diagnosis of SFN in China.

We found that N_2_–P_2_ amplitude varied greatly among subjects, and that N_2_ and P_2_ latency were more reliable parameters to evaluate small nerve fiber function. Different studies have used differing numbers of CHEP stimuli, from as few as 5 to as many as 20 stimuli (Atherton et al., 2007; Chao et al., 2007, 2008, 2010; Casanova-Molla et al., 2011; Haefeli et al., 2014; Lagerburg et al., 2015; Granovsky et al., 2016). In this study, we conducted a pilot study to determine the optimal number of stimuli (3, 5, or 10) at the FA, and found that N_2_ latency/height and P_2_ latency/height did not significantly differ with the number of stimuli. However, N_2_–P_2_ amplitude significantly decreased with an increase in the number of stimuli. Furthermore, 3 stimuli provided the same efficacy as 10 stimuli. Fewer numbers of stimuli were time-consuming, reduced the misery of participants during CHEP test and avoided across site habituation for CHEP test was applied on multiple sites. Therefore, we applied 3 stimuli at each body site in subsequent sessions to establish the normal values, which simplified the CHEP test and made it more efficient. In addition, blink and startle artifacts were carefully monitored and eliminated to ensure the efficacy of the test. The possible pitfalls of reduced stimuli include 3 stimuli may reduce the accuracy of the CHEP test if blink and startle artifacts were not carefully monitored, and larger sample test should be done to further confirm the efficacy of 3 stimuli.

In our study, N_2_ latency/height and P_2_ latency/height were significantly shorter at a baseline temperature of 35°C than at 32°C, and N_2_–P_2_ amplitude and VAS scores were significantly higher at the higher temperature value, which is consistent with the findings of other studies (Kramer et al., 2012a,^2013^; Lagerburg et al., 2015). In four subjects, no recordings were obtained at the LE site at a baseline temperature of 32°C, but were obtained when the temperature was increased to 35°C. Similar observations have been reported in previous studies (Itskovich et al., 2000; Chen et al., 2006; Warbrick et al., 2009; Kramer et al., 2012a; Lagerburg et al., 2015).

Possible explanations for these findings include (1) the higher baseline temperature reduces the time required to reach the peak temperature of 51°C, leading to the activation of a greater number of nerve fibers, and (2) at the higher temperature, the ultra-late C-fiber response does not influence the late Aδ response (Truini et al., 2007; Lagerburg et al., 2015).

We also confirmed the short-term and long-term repeatability of CHEP testing by showing that CHEP parameters did not change significantly with time (baseline vs. 30 min later vs. 1 year later). Most studies have focused on the short-term reproducibility of CHEP stimulation, and found excellent reproducibility (Kramer et al., 2012b; Lagerburg et al., 2015), which is consistent with our findings. Ruscheweyh et al. (2013) examined the long-term (6 months) reproducibility of CHEP, and found that both amplitude and latency changed over time (Ruscheweyh et al., 2013). Possible reasons for this include seasonal differences in skin conductivity and psychological effects (Ruscheweyh et al., 2013). In our study, we included only healthy subjects and excluded seasonal effects by using a time interval of 1 year, and were able to confirm the short- and long-term reproducibility of CHEP.

The correlation of CHEP parameters with age, sex, and body height differs among different studies, and thus, studies with larger sample sizes are required to further investigate this issue.

## Conclusion

We have established the normal values of CHEP parameters in a relatively large Chinese cohort, which facilitated the diagnosis of SFN in China. Only 3 stimuli were required to obtain reliable results on CHEP testing, and this has possibly simplified the examination technique. We found that CHEP parameters changed significantly with baseline temperature, but the short-term and long-term reproducibility of CHEP stimulation was excellent, which is useful in the diagnosis and follow-up of SFN.

## Data Availability Statement

The raw data supporting the conclusions of this article will be made available by the authors, without undue reservation.

## Ethics Statement

The studies involving human participants were reviewed and approved by Ethics Committee of Chinese PLA General Hospital. The patients/participants provided their written informed consent to participate in this study.

## Author Contributions

BS and XH designed the study. BS and ZC conducted the study. BS, HW, and ZC collected and analyzed the data. HW, FC, and FY contributed samples collection and intellectual input. BS drafted and wrote the manuscript. XH revised the manuscript critically for intellectual content. All authors gave intellectual input to the study and approved the final version of the manuscript.

## Conflict of Interest

The authors declare that the research was conducted in the absence of any commercial or financial relationships that could be construed as a potential conflict of interest.

## Publisher’s Note

All claims expressed in this article are solely those of the authors and do not necessarily represent those of their affiliated organizations, or those of the publisher, the editors and the reviewers. Any product that may be evaluated in this article, or claim that may be made by its manufacturer, is not guaranteed or endorsed by the publisher.

## Abbreviations

CHEPs: contact heat evoked potentials
SFN: small fiber neuropathy
IENFD: intraepidermal nerve fiber density
NCS: nerve conduction studies
EMG: electromyography.
